# The causal link between cardiometabolic risk factors and gray matter atrophy: An exploratory study

**DOI:** 10.1016/j.heliyon.2023.e21567

**Published:** 2023-10-31

**Authors:** Vibujithan Vigneshwaran, Matthias Wilms, Nils D. Forkert

**Affiliations:** a Department of Radiology, University of Calgary, Calgary, AB, Canada; b Hotchkiss Brain Institute, University of Calgary, Calgary, AB, Canada; c Department of Pediatrics, University of Calgary, Calgary, AB, Canada; d Department of Community Health Sciences, University of Calgary, Calgary, AB, Canada; e Alberta Children's Hospital Research Institute, University of Calgary, Calgary, AB, Canada

**Keywords:** Normal aging, Gray matter, Causality, Causal discovery, Cardiometabolic risk factors, Medical imaging, UK biobank

## Abstract

Although gray matter atrophy is commonly observed with aging, it is highly variable, even among healthy people of the same age. This raises the question of what other factors may contribute to gray matter atrophy. Previous studies have reported that risk factors for cardiometabolic diseases are associated with accelerated brain aging. However, these studies were primarily based on standard correlation analyses, which do not unveil a causal relationship. While randomized controlled trials are typically required to investigate true causality, in this work, we investigated an alternative method by exploring data-driven causal discovery and inference techniques on observational data. Accordingly, this feasibility study used clinical and quantified gray matter volume data from 22,793 subjects from the UK biobank cohort without any known neurological disease. Our method identified that age, sex, body mass index (BMI), body fat percentage (BFP), and smoking exhibit a causal relationship with gray matter volume. Interventions on the causal network revealed that higher BMI and BFP values significantly increased the chance of gray matter atrophy in males, whereas this was not the case in females.

## Highlights

• Cardiometabolic risk factors are causally linked to gray matter atrophy.
• Body mass index (BMI) and body fat percentage (BFP) showed sex-specific effects.
• Higher BMI and BFP significantly increased the chance of gray matter atrophy in males.

## Abbreviations

DAG: Directed acyclic graph
WHR: Waist to Hip Ratio
BFP: Body Fat Percentage
BMI: Body Mass Index
GMV: Gray Matter Volume
SBP: Systolic Blood Pressure
NOTEARS: Non-combinatorial Optimization via Trace Exponential and Augmented lagRangian for Structure learning
ROC-AUC: Receiver Operating Characteristic - Area Under the Curve
SCM: Structural Causal Modeling
UKBB: UK Biobank

## 1 Introduction

Normal aging is associated with complex structural changes of the brain tissue, for example, due to neuronal death and breakdown of connections between neurons [1]. This so-called atrophy has been associated with a decline in brain function, while regional gray matter volume changes in particular have been linked to impairment of specific brain functions such as cognition [2,3]. However, even among healthy individuals without any known neurological disease within the same age group, the extent of cognitive decline and gray matter atrophy can vary significantly. This variability raises the question of what other factors might contribute to brain atrophy aside from normal aging in healthy subjects.

Several factors have been reported in the literature to be associated with gray matter atrophy, including gender [4], genetic and environmental factors [5], physical activity [6], cigarette smoking [7], and cannabis use [8]. In addition, cardiometabolic risk factors are known to play a significant role in accelerating the aging process of the brain. Fukuda and Kitani [9] found that individuals with medically controlled hypertension have a lower risk of cognitive decline compared to those who are undiagnosed or untreated. Further, research suggests that waist-to-hip ratio (WHR), body mass index (BMI), and body fat percentage (BFP) may also be associated with accelerated brain aging [1,10,11]. Additionally, a growing body of literature indicates that aerobic fitness has a positive impact on various factors related to brain health [12,13]. Nevertheless, it is still an open question whether these cardiometabolic risk factors directly cause brain structural changes or only have a correlation due to a common confounder.

Cardiometabolic disorders are also known to exhibit sex differences [14] and sex-specific links of cardiometabolic risk factors with brain aging and neurodegenerative diseases have been reported [15]. Furthermore, it has been suggested that the so-called brain age gap is influenced by cardiometabolic risk factors in a sex-specific manner [16]. Briefly described, the brain age gap denotes the difference between a person's biological and chronological age, whereas the biological age is typically determined using a machine learning model trained to determine the assumed brain age of an individual based on structural neuroimaging data [17]. However, the causal relationship between cardiometabolic risk factors and gray matter atrophy remains unclear in terms of how it is influenced by sex.

The main problem in identifying true cause-effect relationships is that the available data is primarily observational, allowing correlation-based analyses. However, a statistically significant correlation between two variables does not necessarily indicate a causal relationship between them. Several complex interactions between variables may exist in the context of cardiometabolic risk factors and gray matter atrophy, which may be overlooked in univariate correlational analyses. This limitation underscores the importance of causal investigations in medical and clinical fields. Analyzing causality is a complex challenge, and randomized controlled trials and longitudinal follow-up assessments are generally necessary to effectively identify causal factors and eliminate confounding factors. So far, only a few longitudinal studies have investigated the association between cardiometabolic risk factors and brain atrophy. For example, Tzourio et al. [18] conducted a longitudinal study in elderly subjects, demonstrating a correlation between high blood pressure and cognitive decline. Another recent longitudinal study by Wei et al. [19] found that cardiovascular risk factors are linked to cognitive decline across various cognitive tasks. A potential reason for the scarcity of longitudinal studies is that collecting longitudinal data is a time-consuming process, and in certain cases, conducting a true prospective randomized controlled trial to identify causal links may not be ethically feasible and practically hard to achieve.

Alternatively, causal structure discovery and subsequential intervention methods could address this dearth of randomized controlled trials. Automated causal structure discovery typically starts with a fully connected graph of all known factors that may be causally related and the algorithm then iteratively removes the edges using asymmetry in the relations by solving an optimization problem. The result is a directed acyclic graph (DAG) that optimally represents the causal structure in a dataset [20]. Here, the nodes denote causal factors in the learned DAG, and the directed edges represent the causal relationships. The interested reader can refer to the review by Nogueira et al. [21] for more detailed descriptions.

The DAG obtained through causal discovery serves as a valuable tool for expressing a complex probability distribution in terms of univariate conditional distributions. Simply described, the dataset's joint distribution can be expressed as a product of univariate distributions, where each distribution only depends on its corresponding parent variables in the graph. This representation is commonly referred to as a Bayesian Network. The Bayesian network enables us to simplify joint probabilities in terms of parent-children's relationships, which essentially facilitates causal inference. Furthermore, causal interventions, the process of setting one parent variable to a specific value and quantitatively measuring how it affects its children, can be performed using the do-calculus technique developed by Pearl [22]. The intervention on the causal graph helps to independently measure the effects of each causal factor by accounting for confounding variables. In the context of cardiometabolic risk factors, this allows us to estimate how, for example, a favorable lifestyle change ameliorates gray matter atrophy. Thus far, researchers have employed Bayesian networks for modeling conditional distribution in various domains. Specifically, in neuroscience, Bayesian networks have been used to model post-stroke outcomes [23], cognitive impairment, and Alzheimer's disease [24,25]. More recently, Mouches et al. [26] analyzed the effects of cardiometabolic risk factors on the brain age gap using 2025 participants from the SHIP dataset (Study of Health in Pomerania [27]).

In contrast to the study conducted by Mouches et al. [26], the objective of our work was to conduct a feasibility study that directly examines the causal relationship between cardiometabolic risk factors and gray matter volume, with a focus on identifying age- and sex-specific factors. We specifically chose gray matter volume for our analysis because it has been frequently linked to the decline in cognitive performance during the normal aging process [28]. Additionally, there is a substantial body of neuroimaging research reporting correlations between markers of obesity, such as body mass index (BMI) and waist circumference, and gray matter volume [29,30]. However, there could be multiple intricate interactions between these factors and gray matter atrophy. Thus, the objective of this work is to determine which of these cardiometabolic risk factors demonstrate a direct causal relationship with gray matter volume, and which factors may act as confounders.

This study used quantified normalized gray matter volume, age, sex, WHR, BFP, BMI, smoking, physical activity, and systolic blood pressure data from 22,793 subjects provided by UK Biobank. To analyze the data, we first identified the causal structure in terms of a DAG and then fitted a Bayesian network, which was quantitatively evaluated. Finally, we used the learned conditional distribution table to infer the causal effect of each factor on gray matter volume. This exploratory study demonstrates the usefulness of causal analysis for identifying factors that causally contribute to gray matter atrophy, as well as how the impact of cardiometabolic risk factors on gray matter volume varies based on age and sex.

## 2 Methods

### 2.1 Data

This study uses data from the UK Biobank cohort [31]. UK Biobank (UKBB) has obtained ethical approval from the Northwest Multi-Centre Research Ethics Committee (MREC), and all UKBB participants provided informed consent. MREC approval means that researchers do not require separate ethical clearance. This study retrieved data under application 77,508: Explainable and interpretable machine learning solutions in computational medicine.

The UKBB's T1-weighted structural magnetic resonance imaging (MRI) used a 3D MPRAGE sequence with a 1-mm isotropic resolution and 208 × 256 × 256 mm field of view. Primary brain imaging documentation can be found online (brain_mri.pdf). Our study used the volume of gray matter normalized for head size (UKBB data field: 25,005), which was determined using an image-processing pipeline developed on behalf of UKBB [32].

In the first step, starting with all subjects with available T1-weighted MRI data, participants with diagnosed brain-related disorders based on ICD10 codes (data field 41,202, chapter V - Mental and behavioral disorders and chapter VI -Diseases of the nervous system) were excluded. From the remaining 35,344 participants, we further excluded 291 subjects with a previous stroke (data field: 4056). The sex of participants was retrieved from the genetic sex data field 22,001, and age from the recorded value during the imaging visit (data field: 21,003–2.0). The BMI and BFP values were acquired directly from the dataset using the respective UKBB data fields 21,001 and 23,099. Next, WHR was computed based on waist circumference (data field: 48) and hip circumference (data field: 49) as measured during the imaging visit. We then estimated physical activity by combining the duration of moderate activity per day (data field: 894) and the duration of vigorous activity per day (data field: 914). Two systolic blood pressure readings were taken during the imaging visit (data field: 4080–2.0, 4080–2.1), which we averaged to derive the final value. Smoking status (data field: 20,116) was binarized and set to ‘True’ if the subject is a current/previous smoker. Finally, subjects with NaN values of either BMI, WHR, BFP, or smoking were removed from the table, resulting in a final sample size of 22,793 subjects ranging from 44 to 82 years of age (male = 11,046, female = 11,747).

### 2.2 Causal discovery

Causal inference is a valuable tool that becomes especially powerful when we possess some knowledge about the underlying causal structure. Typically, these causal structures are defined based on expert knowledge and/or common sense. However, in cases where explicit knowledge of the causal structures is lacking, we can employ causal discovery techniques to estimate the corresponding parts of the causal model using available observational data.

Causal discovery involves the challenging task of estimating the causal structure responsible for generating the observed data. This inverse problem lacks a standard method for solving it. To narrow down potential solutions, several assumptions are made during causal discovery, similar to other inverse problems. Four main assumptions are commonly employed: 1. The underlying causal structure can be represented using a directed acyclic graph (DAG), 2. All nodes are independent of their non-descendants when conditioned on their parents, 3. All conditional independences in the true underlying distribution are encoded into the structure, 4. Any pair of nodes in the graph has no common external cause. Building upon these assumptions, various algorithms have been developed to infer the causal structure from observational data, including conditional independence testing methods, greedy search space methods, and techniques exploiting asymmetry in the data. The fundamental concept behind all these approaches is to iteratively refine the graph structure to better capture the relationships within the data. For a comprehensive understanding of these techniques, the article by Glymour et al. [33] provides a thorough review.

### 2.3 Structural causal model

After selecting the factors potentially associated with the gray matter volume, the next step in the analysis was discovering the underlying DAG in an automated fashion. Initial conditions of the DAG were specified from existing domain knowledge, and then the NOTEARS (Non-combinatorial Optimization via Trace Exponential and Augmented lagRangian for Structure learning) algorithm was applied to estimate the graph. When conducting causal discovery with the NOTEARS algorithm, we set two important prior conditions. First, we explicitly define that the age and sex variables are not influenced by any other factors present in the model. Second, we defined that gray matter volume cannot have any children in the causal structure. By setting these prior conditions, we provide guidelines to the algorithm for determining causal relationships and constructing the causal structure. The remainder of the graph structure was discovered using the NOTEARS algorithm [34].

The state-of-the-art NOTEARS algorithm restates structure discovery as a continuous optimization problem over real matrices, which can be efficiently solved using existing numerical techniques. Therefore, each factor in the graph was assigned to a continuous, categorical, or binary schema. Gray matter volume, age, BMI, BFP, systolic blood pressure, and WHR were defined as continuous variables, while sex and smoking were defined as binary variables. Next, the continuous variables were normalized by subtracting the mean of each variable and a division by the standard deviation. This step ensured that all features were assessed equally, irrespective of their absolute values. Next, the graph's structure was optimized using the NOTEARS algorithm, which resulted in a cyclic graph. Finally, weaker edges in this graph, which denote weaker associations between variables, were iteratively removed until the graph was reduced to a DAG.

### 2.4 Bayesian network

In the next step, a Bayesian network was modeled by encoding the observational conditional probability distribution of the data. A Bayesian network is a probability model where each variable is conditioned on its parents in the graph. In this network, nodes represent variables, and directed edges depict the conditional relationship. The nodes d-separated in the graph are conditionally independent of the given variables. To perform d-separation, a set of variables that are believed to influence the relationship between the two variables of interest are identified. Suppose any path between the two variables is not blocked by one of these influence variables, then the two variables are said to be d-separated and conditionally independent. Thus, a Bayesian network simplifies a complex joint distribution in terms of conditional probabilities as follows:

$$p\left(x_{1},x_{2},\ldots ,x_{m}\right)=\prod_{k=1}^{m}p\left(x_{k}|\;x_{parent\left(k\right)}\right)$$

Typically, the edges in a Bayesian network do not imply causation but only the association/correlation between variables. However, because a causal DAG has already been discovered, the modeled Bayesian network can be considered a causal network [34]. It is important to note that all continuous variables in the data were discretized prior to the Bayesian modeling step because encoding continuous values would result in an intractable network. Practically, each continuous variable was divided into four states using the univariate distribution's 25th, 50th, and 75th percentile (see Table 1). Next, Bayesian parameter estimation was used to model the conditional probability distribution with a Dirichlet distribution as the Bayes prior [35].

**Table 1:** Statistics of the discretized factors in the graph. BMI: Body mass index, BFP: Body fat percentage, WHR: Waist-Hip ratio, and SBP: Systolic blood pressure.

Variables	State	Range	Males	Females
Age (years)	0	<58	2450	3113
	1	58–64	2376	2913
	2	64–70	2998	3194
	3	≥70	3222	2527
BMI (kg/m^2^)	0	<23.3	1785	3913
	1	23.3–25.6	2883	2815
	2	256–28.3	3314	2382
	3	≥28.3	3064	2637
BFP	0	<24.7	5076	622
	1	24.7–30	3976	1701
	2	30–36.2	1784	3924
	3	≥36.2	210	5500
WHR	0	<0.8	235	5459
	1	0.8–0.87	1693	4003
	2	0.87–0.93	3957	1729
	3	≥0.93	5161	556
Physical activity (minutes/week)	0	<40	2563	2841
	1	40–70	2631	2959
	2	70–120	2736	2667
	3	≥120	3116	3280
SBP (mmHg)	0	<125	1817	3809
	1	125–137	2774	2826
	2	137–150	3238	2572
	3	≥150	3217	2540
Smoking	0	–	6793	7941
	1	–	4253	3806
Gray matter volume (ml)	0	<761.5	3903	1795
	1	761.5–793.3	3108	2590
	2	793.3–826.5	2498	3200
	3	≥826.5	1537	4162

After the generation of the Bayesian network, it was employed to predict the probability of each state of a variable conditioned on all its parents. This inference technique was used to evaluate the quality of the fitted model. First, every variable $x_{i}$ was conditioned on its parents, and the most probable state of $x_{i}$ was inferred. Next, the inferred and actual states were compared, and the classification accuracy was measured using the area under the receiver operating characteristic curve (ROC-AUC) using 10-fold cross-validation. Finally, the Bayesian network with the highest ROC-AUC was selected from the cross-validation step, and the entire dataset was used to fit the Bayesian network for refinement.

## 3 Results

**Fig. 1:**
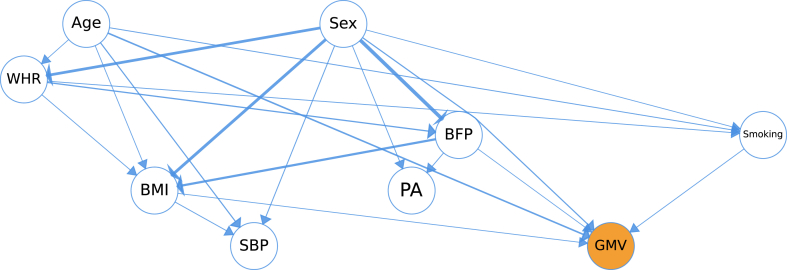
Discovered causal structure from the data. The nodes display the factors, the directed edges indicate causal relationships, and edge thickness encodes the weight (stronger = thicker). BMI: Body mass index, BFP: Body fat percentage, WHR: Waist-Hip ratio, PA: Physical activity, GMV: Gray matter volume, and SBP: Systolic blood pressure.

Fig. 1 shows the discovered causal structure of age, sex, the cardiometabolic risk factors, and GMV. In this DAG, the nodes display the variables, while the directed edges indicate causality. Thus, the parent-children relationship represents cause and effect. In addition, the edge thickness indicates how strongly each parent influences their child (stronger = thicker).

It can be seen from the figure that five factors (age, sex, smoking, BMI, and BFP) affected gray matter volume. The absolute values of the weights of the causal factors are as follows: Age = 0.58, Sex = 0.33, Smoking = 0.12, BMI = 0.10, and BFP = 0.07. WHR did not causally influence gray matter atrophy. However, it influenced BMI and BFP, thus indirectly affecting GMV. Furthermore, the graph shows that the duration of physical activity was caused by sex and BFP and did not affect GMV directly. In comparison, age and sex were predominantly responsible for the GMV changes, while a moderate association between cardiometabolic risk factors (BMI, BFP, smoking) and GMV can be noticed. Interestingly, both SBP and GMV, were caused by cardiometabolic risk factors, yet they did not influence each other directly. This leads to the finding that although there was a correlation between SBP and GMV, they were causally independent, as confounding variables (cardiometabolic risk factors) explained them sufficiently. Furthermore, the thicker edges originating at the sex node show that BMI, BFP, and WHR were biased based on sex. In general, the graph discovered closely aligns with the existing clinical knowledge [1,4,10,16].

Once the model was fitted to the UKBB data, the validity of the Bayesian network model was evaluated using 10-fold cross-validation. The mean value of the ROC-AUC and standard deviation were calculated for each child as follows: SBP = 0.65 (0.003), BMI = 0.81 (0.004), smoking = 0.68 (0.012), PA = 0.55 (0.008), and GMV = 0.75 (0.005). Compared to our previous study [26], these results show an increase in overall accuracy. This improvement can be attributed to the larger sample size from the UKBB, which has enhanced the model's ability to identify causal relationships and perform more robust Bayesian analyses. Therefore, we are confident that the result of this validation ensured that the data distribution was sufficiently encoded into the model.

**Fig. 2:**
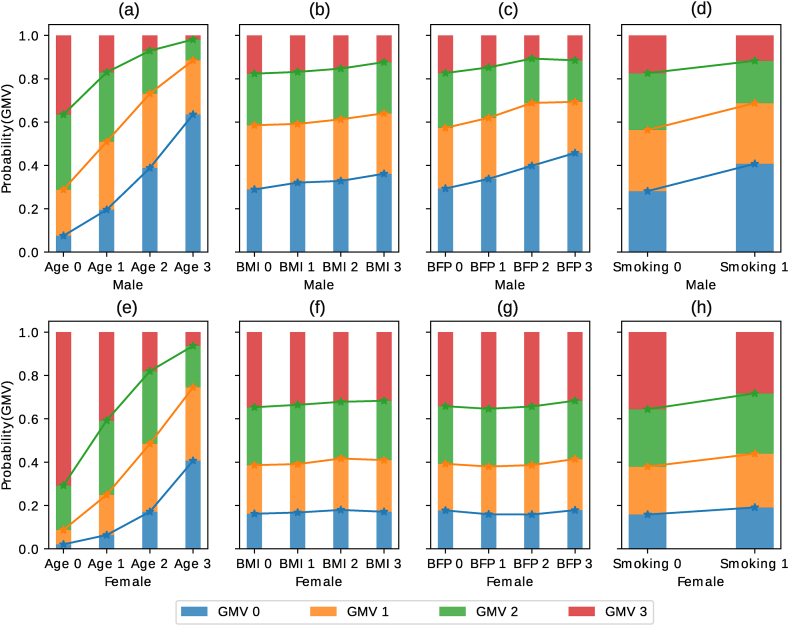
Sex-specific effects of causal factors on gray matter volume, where the subfigures labeled (a), (b), (c), and (d) depict the individual effects of Age, BMI, BFP, and Smoking on males. Likewise, (e), (f), (g), and (h) represent the corresponding effects on females. Here BMI: Body mass index, BFP: Body fat percentage, and GMV: Gray matter volume. The x-axis shows grouped parent factors, where Age 0:<58, Age 1:58–64, Age 2:64–70, and Age 3: ≥ 70 (for other groups, see Table 1). The y-axis shows the probability of each GMV group, where GMV 0 is low and GMV 3 is high. For instance, the Male - Age 3 group has a higher probability of GMV 0 than GMV 3.

During the intervention step, the marginal probability of GMV conditioned on age, BMI, and smoking status were calculated for males and females separately (Fig. 2). The results of this analysis suggest that the female population has a higher probability of larger normalized GMV compared to the same group of males. The figure further indicates that aging was the principal cause of gray matter atrophy. The likelihood of having lower GMV was 58 % for males and 38 % for females in age group 3 ( ≥ 70 years). This implies that older males with higher BMI demonstrated reduced GMV compared to older females with higher BMI. Furthermore, the figure shows how smoking impacted the gray matter volume in both sexes. It is interesting to note that BMI and BFP significantly affected the aged male population but not the aged female population. A higher BMI increased the chance of gray matter atrophy in males, whereas BMI and BFP did not affect GMV in females.

**Fig. 3:**
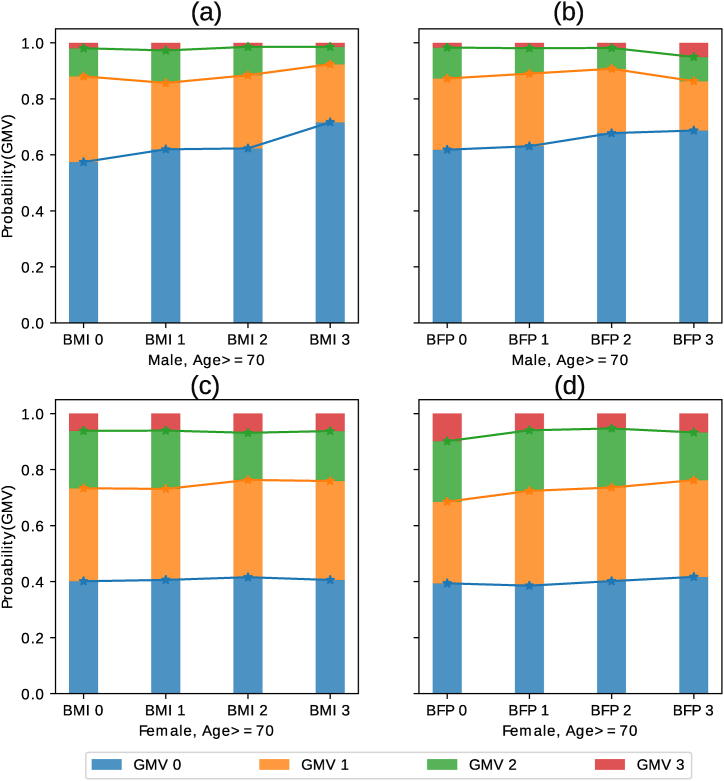
Sex-specific effects of the body mass index (BMI) and body fat percentage (BFP) on the aged population. Subfigures (a) and (b) display the effects of BMI and BFP on older males, while subfigures (c) and (d) illustrate the effects on older females. The x-axis shows grouped parent factors of BMI and BFP (see Table 1). The y-axis shows the probability of each gray matter volume (GMV) group, where GMV 0 is low and GMV 3 is high. For instance, the Male – Age ≥ 70 – BMI 3 group has a higher probability of GMV 0 than GMV 3.

Next, multiple interventions were introduced in the Bayesian network, and it was determined how BMI and BFP affect the elderly population specifically. The sex-specific effect of the BMI and BFP on the aged population was measured using the following two interventions: p(GMV|do(sex=male,age=high) and p(GMV|do(sex=female,age=high). It can be seen from Fig. 3 that the older male population is more susceptible to increased BMI and BFP than older females.

**Fig. 4:**
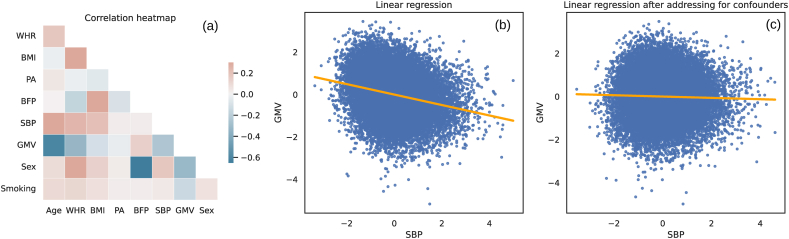
Correlation analysis on the observational data. **(a)**: correlation coefficients of each pair of variables, (b) standard linear regression plot, and (c) linear regression plot after addressing for the confounders Age and BMI. BMI: Body mass index, BFP: Body fat percentage, WHR: Waist-Hip ratio, PA: Physical activity, GMV: Gray matter volume, and SBP: Systolic blood pressure.

In addition to these experiments, we measured the correlation coefficients of each pair of variables. It can be seen from Fig. 4 that SBP and GMV show a strong negative correlation. However, the correlation between SBP and GMV decreased significantly after addressing the confounders (Age and BMI), which were identified using the causal analysis. This experiment shows the importance of addressing confounders in univariate analysis and underpins the importance of causality-based techniques.

## 4 Discussion

This study demonstrates how causal analysis can effectively identify and measure underlying causal structures when a randomized controlled trial is not feasible. Specifically, we identified the causal linkage between cardiometabolic risk factors and GMV and modeled a Bayesian network to explain the data distribution in the UKBB cohort. This feasibility study shows that considering the causal relationships between age, sex, and cardiovascular risk factors enables us to model as well as explain complex relationships between them. Additionally, these methods can be easily applied to other neuroimaging clinical issues.

Using causal analysis, we observed that age, sex, BMI, BFP, and smoking affect the gray matter volume directly. This finding is generally in line with correlation-based studies of cardiometabolic risk factors and the brain age gap [1,10,11,36]. Among the causal factors identified, age accounted for a substantial proportion of the effect, followed by sex, BMI, smoking, and BFP.

We found no significant links between SBP and GMV. Within this context, it needs to be highlighted that the SBP and GMV had common parents, including BMI, age, and sex. These confounding factors could have caused the correlation between SBP and gray matter atrophy observed in correlation-based studies such as [10,37], and as also demonstrated in our standard correlation analyses. In line with our findings, Cox et al. [38] reported that accounting for confounding variables leads to a reduced effect of the pulse pressure on gray matter measurements. In another related study, Siedlinski et al. [39] discovered a connection between blood pressure and a specific set of imaging-derived characteristics using Mendelian randomization. In future work, it would be interesting to expand our approach to different regions of the brain and investigate how this may affect the specific causal findings.

By employing interventions on the causal graph, we were able to isolate the effects of each causal factor on GMV while considering confounding variables. Specifically, when examining sex-specific interventions, we found that older males with higher BMI exhibited smaller normalized GMV in comparison to older females with higher BMI. It is worth noting that previous studies have also observed sex-specific effects in various gray matter regions. For example, in the study conducted by Taki et al. [4], gray matter regions, including the basal aspect of the temporal and occipital lobes (including the fusiform gyrus, inferior occipital gyrus, parahippocampal gyrus, and lingual gyrus), exhibited more pronounced (age × sex) interactions compared to other brain regions. In a recent study conducted by Subramaniapillai et al. [16], the potential variations in the associations between white matter BAG and BMI, WHR, BFP, and APOE4 status in males and females were investigated. The findings of that study indicated sex differences in these associations, with males exhibiting stronger positive relationships between BAG and all three cardiometabolic risk factors (BMI, WHR, and BFP) compared to females.

The exact causes of these sex differences remain speculative and necessitate additional investigation. However, several factors could potentially contribute to this finding, including variances in hormone levels, differences in body composition, or intricate interactions between sex-related factors and brain structure. Subramaniapillai et al. [16] proposed that considering the impacts of both chronological and endocrine aging may lead to a better understanding of sex-specific brain aging effects.

This study has a few limitations that should be highlighted. The main limitation was the assumption that all confounding variables were available during the causal discovery step. It is essential to list all confounders for the NOTEARS causal discovery algorithm so that the discovered graph can explain the causal relationship accurately. We excluded certain risk factors associated with gray matter atrophy, such as cholesterol and diabetes, due to skewed data and the goal of maintaining a simple causal structure in our feasibility study. In future work, we plan to explore the impact of additional variables, including cholesterol, diabetes, education level, and antihypertensive medications, which could potentially alter the causal discovery results.

In the context of our study, imaging protocols were similar across the study population since this data was from the UKBB so that an imaging-based bias can be excluded. Moreover, since the demographic distribution of UKBB is predominantly UK Caucasians, the discrepancies between internal factors, including genetics and race, may be rather low. Nevertheless, since our analyses were based on observational data, the typical limitations of cross-sectional studies also apply to this work. Performing causal analyses on longitudinal data and encoding expert field knowledge could, therefore, potentially improve the accuracy of causal discovery [40]. Longitudinal data of a subset of the subjects are currently being made available in UKBB so that analyses of this data may be possible in future work.

Another limitation of this work is the discretization of the data for Bayesian network modeling, which decreases the resolution of the data. Structural causal modeling (SCM), which describes a variable $x_{i}=f\left(pa\left(x_{i}\right),e_{i}\right)$ as a function of its parent $pa\left(x_{i}\right)$ and unobserved exogenous noise $e_{i}$, could be applied to overcome this problem in future work. Moreover, SCMs also enable even more advanced causal analyses, including the estimation of counterfactuals. In contrast to our interventions, which perform at the population level, counterfactuals answer the question of “what would have happened if” at an individual level. Recently, there have been several deep-learning-based techniques developed to model SCMs [41,42]. However, these techniques typically require a predefined causal graph or a causal structure discovery algorithm before modeling the probabilities. Since the current feasibility study has provided satisfactory results, we plan to combine NOTEARS and these techniques to model UKBB data in the future.

In conclusion, this work demonstrates the advantages of performing a causal analysis on observational data using interventional queries. Using this technique, we can independently measure the effects of each causal factor, which is otherwise challenging. We identified direct causes of gray matter atrophy and showed how these factors varied with sex and age. Sex-specific gray matter differences in the aged population emphasize that these two groups should be treated separately in clinical settings. Furthermore, the techniques applied in this study can easily be transferred to other domains. For example, the same analyses could be applied to white matter hyperintensities, or the association between specific brain regions and cognitive behavioral changes can be assessed.

## Funding

This work was supported by the 10.13039/501100001804Canada Research Chairs program and the River Fund at 10.13039/100012260Calgary Foundation.

## Data availability statement

This research has been conducted using the UK Biobank Resource under Application Number 77508. The data used for all the analyses in this study are available at the UK Biobank. The procedure to retrieve the data is provided here. The causal discovery algorithm and Bayesian network modeling are available through the package CausalNex (https://causalnex.readthedocs.io/en/stable/index.htmlhttps://causalnex.readthedocs.io/en/stable/index.html).

## CRediT authorship contribution statement

**Vibujithan Vigneshwaran:** Conceptualization, Data curation, Formal analysis, Investigation, Visualization, Writing – original draft. **Matthias Wilms:** Conceptualization, Project administration, Supervision, Writing – review & editing. **Nils D. Forkert:** Conceptualization, Funding acquisition, Project administration, Resources, Software, Supervision, Writing – review & editing.

## Declaration of competing interest

The authors declare that they have no known competing financial interests or personal relationships that could have appeared to influence the work reported in this paper.
